# Fulleropeptide esters as potential self-assembled antioxidants

**DOI:** 10.3762/bjnano.6.107

**Published:** 2015-04-27

**Authors:** Mira S Bjelaković, Tatjana J Kop, Jelena Đorđević, Dragana R Milić

**Affiliations:** 1 Institute of Chemistry, Technology and Metallurgy, Center for Chemistry, University of Belgrade, Njegoševa 12, P.O. Box 473, 11001 Belgrade, Serbia; 2 Faculty of Chemistry, University of Belgrade, Studentski trg 12–16, P.O. Box 51, 11158 Belgrade, Serbia

**Keywords:** antioxidant activity, cyclic voltammetry, FOX assay, fulleropeptide esters, scanning electron microscopy

## Abstract

The potential use of amphiphilic fullerene derivatives as a bionanomaterial was investigated by cyclic voltammetry (CV), scanning electron microscopy (SEM), and the ferrous ion oxidation–xylenol orange (FOX) method. Despite the disrupted delocalization of the π-electronic system over the C_60_ sphere, its antioxidant capacity remained high for all twelve derivatives. The compounds expressed up to two-fold and 5–12-fold better peroxide quenching capacity as compared to pristine C_60_ and standard antioxidant vitamin C, respectively. During precipitation and slow evaporation of the solvent, all compounds underwent spontaneous self-assembly giving ordered structures. The size and morphology of the resulting particles depend primarily on the sample concentration, and somewhat on the side chain structure.

## Introduction

Their highly π-conjugated, spherically shaped, hydrophobic character and their unique physicochemical, electronic and magnetic properties make fullerenes attractive building blocks for chemical modifications, providing at the same time novel opportunities for developing diverse scientific fields, particularly in materials science [1], supramolecular chemistry [2], and medicinal chemistry [3–4]. The derivatization of fullerene with peptide units substantially modifies its original properties, rendering them particularly interesting for structural studies [5] and biological applications [6–7]. Since the first reported synthesis of a fulleropentapeptide by Prato and co-workers [8] in 1993, many examples of hybrids in this field have been described, which has expanded research in different directions.

The functionalized fullerenes are able to self-assemble into a plethora of supramolecular structures, such as spheres, nanotubes, vesicles, rods, nanowires, and nanofibers [9–11]. Also, formation of diverse morphologies of self-assembled fullerene derivatives under different external conditions has also been the subject of interest of Nakanishi's research group [12–13]. Although the morphological characterization of the self-organized fulleropeptide nanoparticles has not been extensively studied thus far, the formation of ordered superstructures under controllable conditions through the process of self-assembly has been observed. Here, cryo-transmission electron microscopy [14] and SEM [15] experiments on aqueous solutions of fullerene-based amino acids and peptides confirmed their strong aggregation behavior and formation of spherical and ellipsoidal clusters.

In the area of biological applications, a wide range of activities of fullerene–peptide conjugates has been studied [3,7,16]. Fulleropeptides synthesized by Prato's research group [17] showed a good bacteriostatic activity against Gram-positive bacterium *S. aureus* making it interesting for potential antimicrobial chemotherapeutics. Recently, Keller et al. [18] reported the synthesis of fullerenedihydropyrrole cationic peptides, which did not show antibacterial activity. Neuroprotective and antioxidant properties are based on the fact that fullerene derivatives possess an extended π-bond system, with high electron and free-radical species affinities. Water-soluble fullerene–alanine adducts were tested as cytoprotective agents showing high effectiveness for removing the reactive oxygen species, such as superoxide anions and hydroxyl radicals [19–20]. The study of the penetration of fulleropeptide nanoparticles through skin represents a major contribution to the development of carriers for biomolecules [21]. Higashi and co-workers have reported the aggregation properties and a high superoxide scavenging activity of fullerene–poly(Glu)peptide nanoparticles as self-assembled structures [22]. Fullerene C_60_ and fulleropyrrolidine derivatives showed significant antioxidant capacity to remove hydroxyl and superoxide radicals when incorporated into liposomes [23].

**Figure 1:**
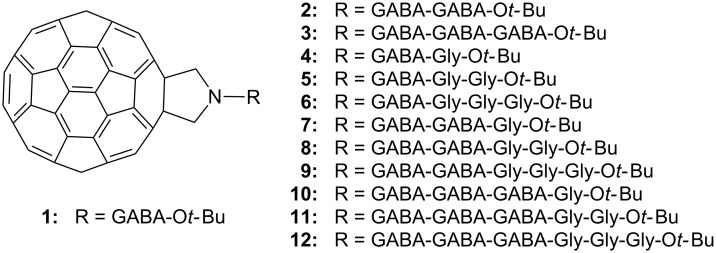
Structures of studied fullereropyrrolidinic (Fp) ester **1** [24] and Fp–peptides **2–12** [27].

With respect to the design and synthesis of fulleropyrrolidine-based hybrids, we previously showed the synthesis of fullerene–steroid esters [24] and triple hybrids consisting of fullerene, peptide, and steroid units [25], which possess in vitro antioxidant activity. Additionally, morphological characterization of self-organized structures of fullerene–steroid [26] and fullerene–peptide–steroid hybrids [25] in solution and in the solid state was studied by SEM. In our previous work [27], we described the synthesis and provided a thorough spectral characterization of a series of fulleropeptide *tert*-butyl esters (**2**–**12**, Figure 1) consisting of two aliphatic amino acids, γ-aminobutyric acid (GABA) and glycine (Gly), using fulleropyrrolidinic ester **1** as a starting compound. In continuation of our interest in the chemistry of fullerene–peptide hybrids, we assumed that their further characterization could contribute to a better understanding of the synergy of the molecular subunits, as well as to potential applications in the field of new bionanomaterials. Consequently, here we present the results of the electrochemical, in vitro antioxidant and morphological investigations of compounds **1**–**12**.

## Results and Discussion

Cyclic voltammetry (CV) of fullerene derivatives plays a relevant role in the characterization of their electronic properties and potential applications [28]. The electrochemical investigation of fulleropyrrolidine esters **1**–**12** by CV was carried out in dimethylformamide (DMF) at room temperature with tetrabutylammonium perchorate (TBAP) as the supporting electrolyte and a ferrocene/ferrocenyl couple (Fc/Fc^+^) as the internal redox standard. The observed half-wave reduction potentials (*E*_1/2_) of the studied compounds together with those of pristine C_60_ (as a reference compound [29]) are listed in Table 1.

**Table 1:** Half-wave reduction potentials vs Fc/Fc^+^ (0.53 V vs Ag/Ag^+^ in DMF) of fullerene esters in DMF containing 0.1 M TBAP as the supporting electrolyte.

Compound	*E*_1/2_ (V)			

	1st	2nd	3rd	4th

**1**	−0.94	−1.40	−2.06	−2.57
**2**	−0.94	−1.39	−2.05	−2.59
**3**	−1.01	−1.39	−2.06	−2.58
**4**	−0.99	−1.38	−2.04	−2.58
**5**	−0.92	−1.38	−2.08	−2.60
**6**	−0.98	−1.40	−2.06	−2.60
**7**	−0.94	−1.39	−2.06	−2.62
**8**	−0.99	−1.38	−2.04	−2.57
**9**	−0.98	−1.36	−2.02	−2.52
**10**	−0.94	−1.39	−2.04	−2.54
**11**	−1.00	−1.39	−2.03	−2.56
**12**	−0.96	−1.39	−2.07	−2.61
C_60_^a^	−0.77	−1.25	−1.84	−2.38

^a^Half-wave reduction potentials from [27] and recalculated according to the Fc/Fc^+^ value.

The voltammetric behavior of the compounds is characterized by the presence of four reversible, one-electron reductions, all attributable to the 58 member π-electron system of fulleropyrrolidinic subunit, similar to that found for other fulleropyrolidine monoadducts [30]. As a consequence of the fullerene [6,6]-double bond saturation (after Prato's reaction), all four registered half-wave potentials were cathodically shifted by 110–240 mV relative to pristine C_60_, and appeared at approximately −1.0, −1.4, −2.0, and −2.6 V vs Fc/Fc^+^ (1st to 4th, respectively).

**Figure 2:**
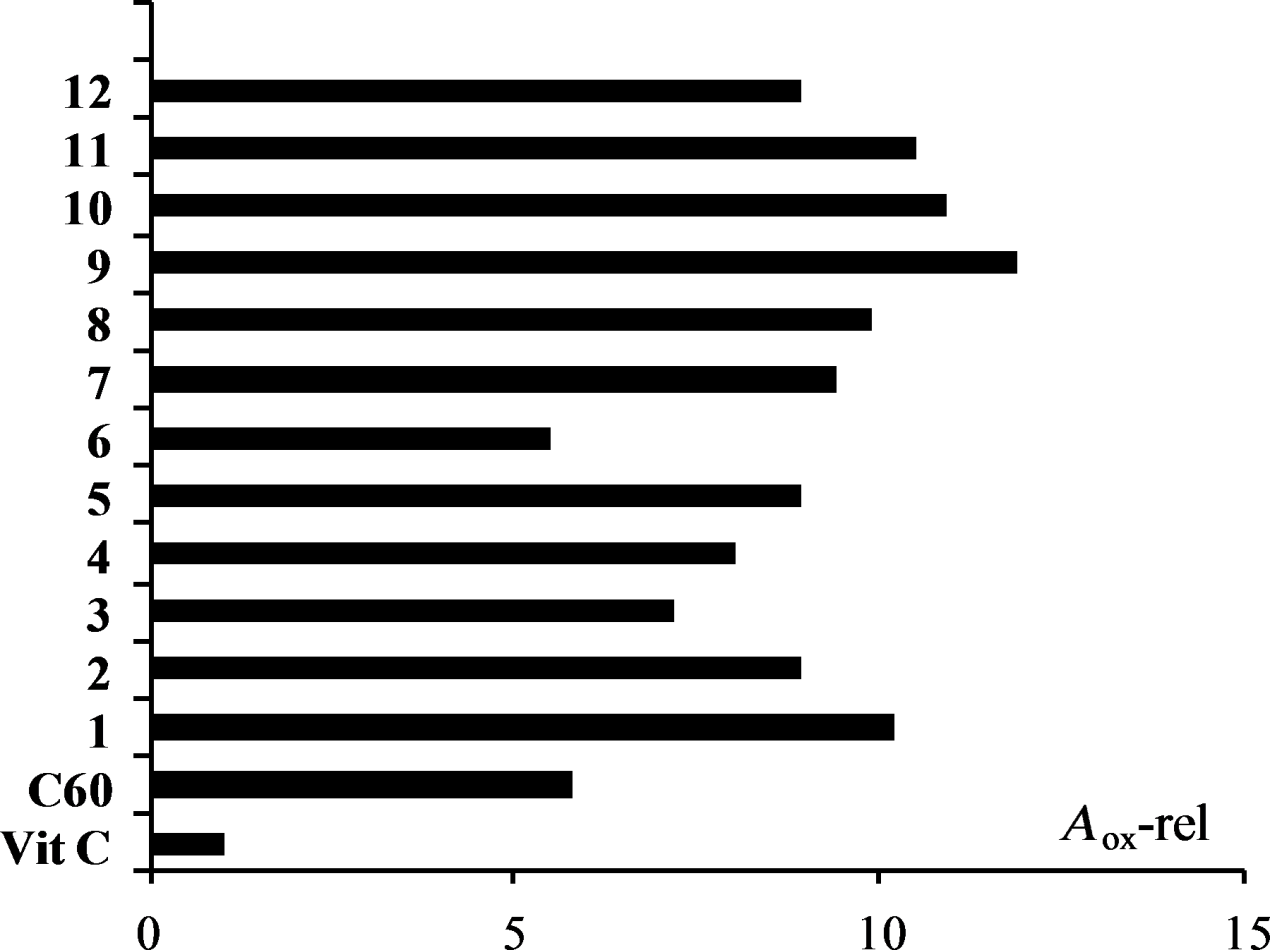
Comparison of the relative antioxidant activities (*A*_ox_-rel) of the tested compounds (**1–12**) and C_60_ with regard to vitamin C (*A*_ox_-rel = 1) measured by FOX assay.

Although the fullerene functionalization resulted in the decrease of the electron affinity, the π-electron system was not dramatically disrupted, thus, it could be expected that the carbon core in the studied compounds retained its antioxidant capacity. The antioxidant activity of 12 fullerene esters (as water soluble fullerosomes, obtained by liposome formation with soybean lecithin [31]) was determined by FOX antioxidant assay [32], using vitamin C and fullerene C_60_ as reference compounds. The FOX assay is based on the oxidation of ferrous to ferric ions in the presence of hydroperoxides in acidic media and subsequent complex formation with xylenol orange (XO), monitored using spectrophotometry by measuring the absorbance at 560 nm (*A*_560_). In the presence of an antioxidant compound, the *A*_560_ decreases due to hydroperoxide quenching. The antioxidant capacity of the compounds studied, expressed as an inhibitory effect toward oxidation, was evaluated by monitoring the formation of an Fe^3+^–XO complex. The experiments were performed by incubating the aqueous solution of *tert*-butyl hydroperoxide (TBHP), tested compounds (0.002 mg/mL) and FOX reagent, followed by the *A*_560_ measurement. The results of the direct antioxidant capacity are expressed as the percentage of consumed TBHP (Δ%) (see Experimental section, Table 2), demonstrating the level of peroxide consumed. Recalculations provided results of molar activities and expressing them in comparison to vitamin C afforded values of the relative antioxidant activity (*A*_ox_-rel), as presented in Figure 2. As can be seen, the fullerene esters showed much higher capability to decrease the level of peroxides in solution compared to the standard antioxidant agent, vitamin C. All the tested fullerene esters expressed five- to twelve-fold higher antioxidant capacity compared to vitamin C, and similarly to C_60_ (compound **6**) with an up to two-fold better performance. It was shown that derivatization of fullerenes with peptide units enhances their solubility [27] and, consequently, their essential antioxidant capacity, which is mainly attributed to the electron-accepting properties of the fullerene unit.

**Figure 3:**
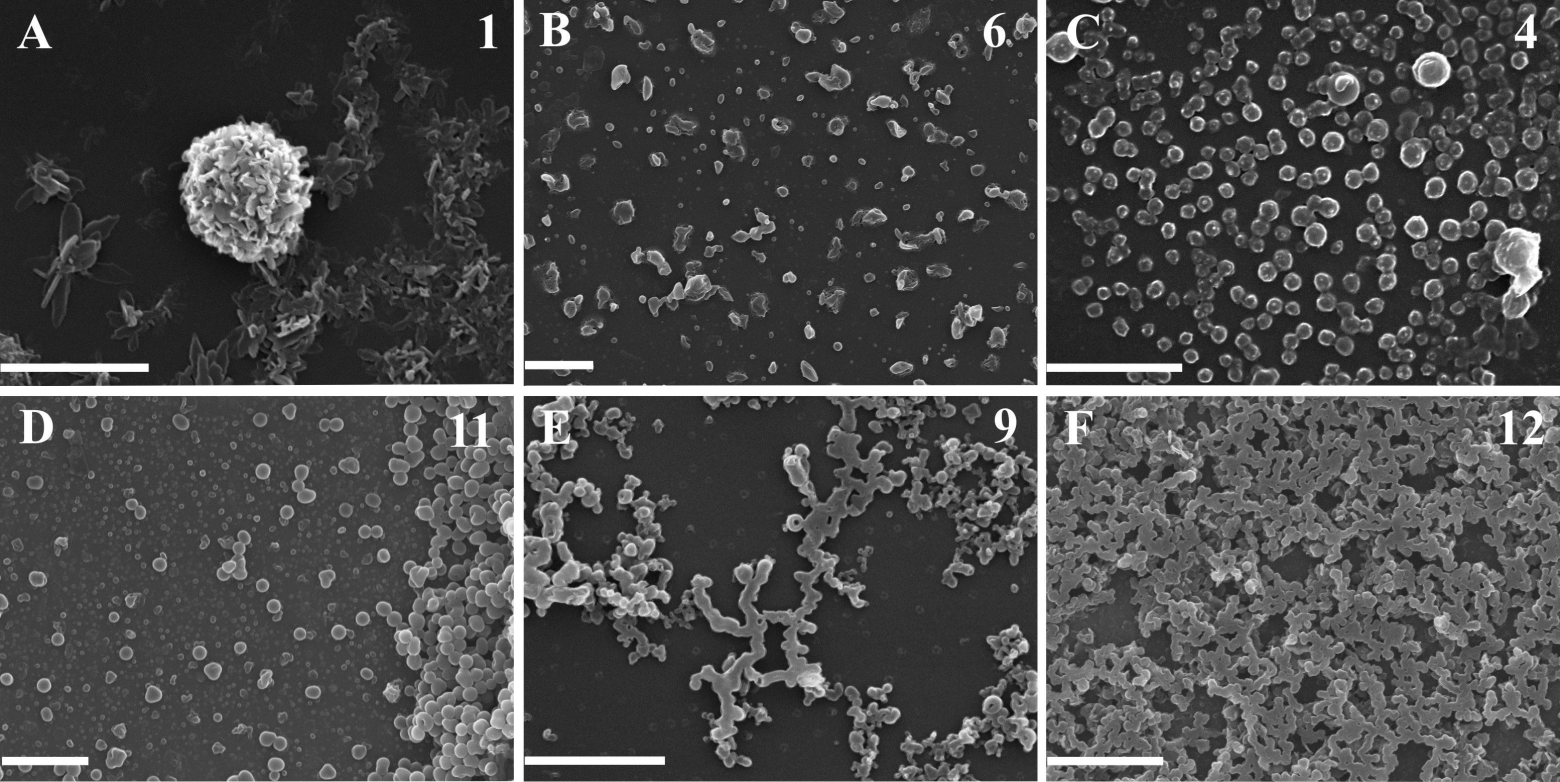
Selected SEM images of flower-shaped, self-organized particles of the parent ester **1** (A), and different fulleropeptide self-assemblies. (B) Isolated, curled microsheets of **6**. (C, D) Isolated, flat, spherical particles of **4** and **11**. (E, F) Networks of spherical particles of **9** and **12**, all prepared from 5:1 PhMe/MeOH (v/v) on a Si substrate after evaporation of a 1 mM solution. Scale bars correspond to 5 μm.

The self-assembly characteristics of fulleropeptide esters **2**–**12** were studied by SEM on the samples prepared by a drop–drying process [2] and on the solid samples. In order to examine the influence of the peptide chain on the self-assembly properties, the aggregation behavior of the parent Fp–GABA ester **1** (the control compound without the peptide moiety) was also included in this investigation. The SEM study on the control compound was reported and showed that the best-ordered, self-organized structures are obtained in a binary solvent mixture, 5:1 PhMe/MeOH [25]. The morphological features of each of the tested fullerene derivatives were investigated under the same conditions, using dried samples obtained from dilute solutions by slow evaporation in a 5:1 PhMe/MeOH mixture (10 µL, 1 mM) on a Si substrate at room temperature. The ester **1** arranged into a flower-shaped, hierarchically ordered architecture of curled leaf-like particles with diameters of up to 5 μm (Figure 3A) [25]. Figure 3B–F shows selected representative examples of SEM micrographs of typical fulleropeptide self-assembled structures. Additionally, SEM images of self-organized structures of all the investigated compounds are shown in Figure S1 (Supporting Information File 1). Two types of rounded particles (flat and curled), originating from spontaneously formed, self-organized spherical nanoparticles, were the dominant structures observed among all the investigated fulleropeptides (Figure 3).

**Figure 4:**
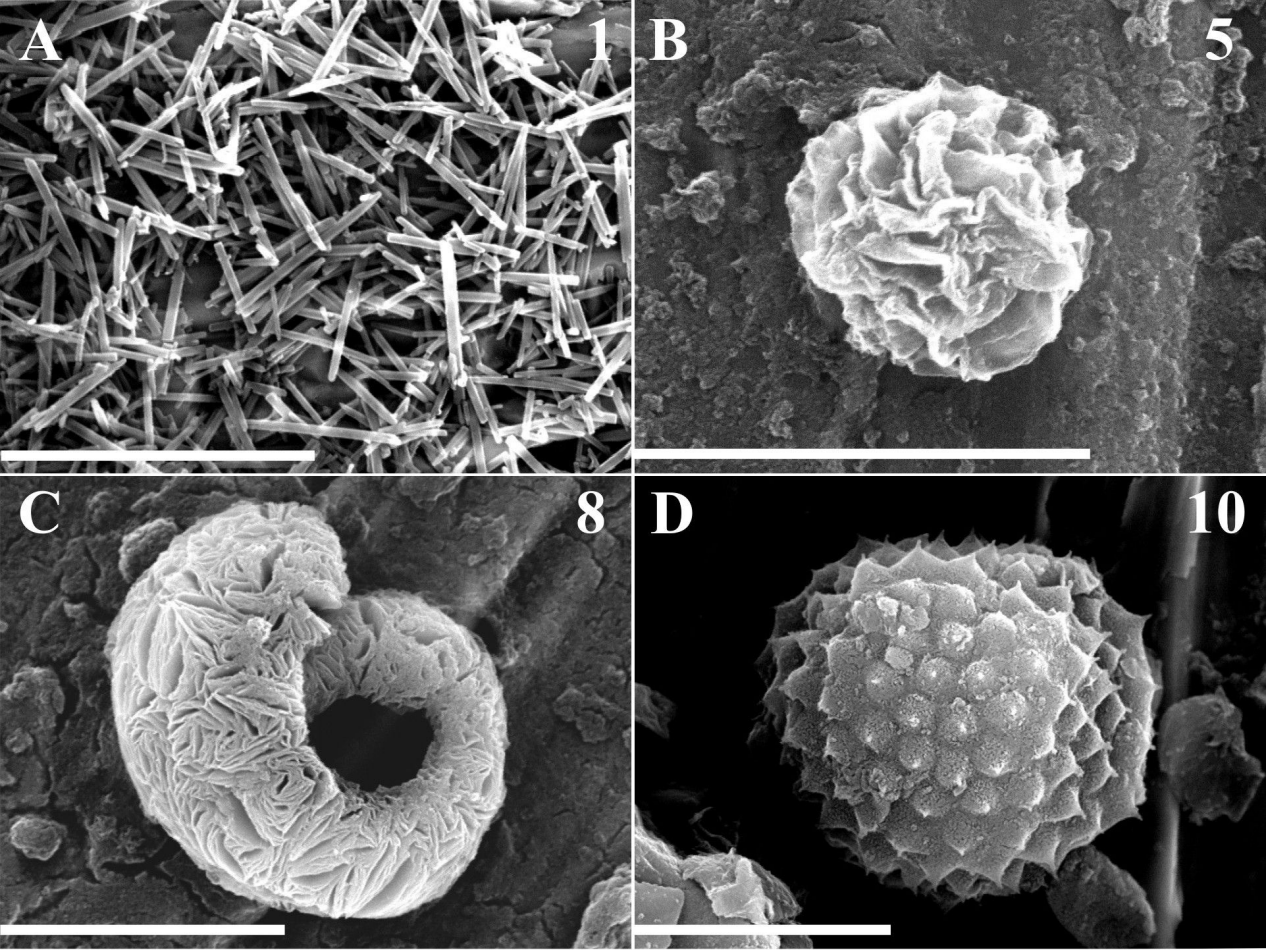
Representatives of the SEM images of the self-organized rods of Fp–GABA ester **1** (A) and fulleropeptides self-assembled particles: flower (B, **5**), spiral-shaped object (C, **8**), and artichoke-shaped object (D, **10**) prepared from the solid samples deposited on a brass substrate. Scale bars correspond to 10 μm.

**Figure 5:**
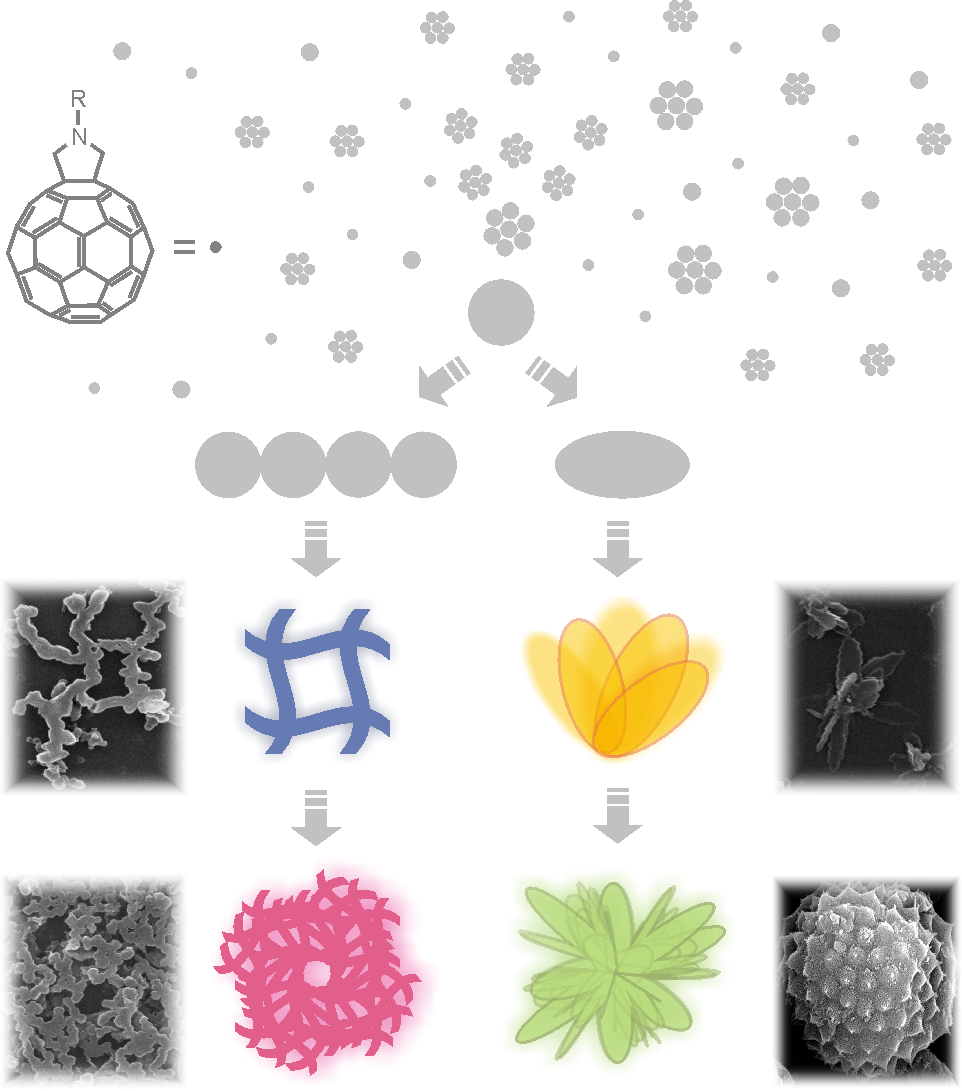
Schematic illustration of the proposed formation of different self-assembled microstructures starting from the fulleropeptide spherical nanoparticles.

The examined GABA containing homopeptides **2** and **3**, tripeptide **7**, and the tetrapeptides **6**, **8**, and **10** all prefer to form similar isolated, curled microparticles as final assemblies with dimensions up to 3 μm (Figure 3B (**6**)). SEM studies revealed that the other five fulleropeptides, **4**, **5**, **9**, **11**, and **12**, self-assembled into mainly individual, flat-spherical nanoparticles of various sizes (Figure 3C,D) or into the network-type structures (Figure 3E,F). It should be noted that the heteropeptides **9**, **11**, and **12** had the increased tendency to form fused or stacked small spherical particles with a flat morphology, giving branched, straight aggregates as the start of network formation (Figure 3E, fulleropeptide **9**), and finally, a complete network of spherical particles (fulleropeptide **12** at a five-fold higher concentration, Figure 3F). Additionally, SEM micrographs (Supporting Information File 1, Figure S1, insets) have clearly revealed network-like structures of three fulleropeptide esters, **9**, **11**, and **12**, obtained at this concentration, indicating the morphology dependence of the investigated compounds on the peptide concentration. It was shown that the longer peptide chain facilitates the formation of a network structure as a consequence of stronger, arranged interparticle associations. The solid samples of the control compound **1**, obtained by precipitation from a CHCl_3_/CS_2_ solution with solvents of different polarity (i.e., MeOH, Et_2_O or hexane, on a brass substrate), gave well-organized particles only with the polar solvent, MeOH, indicating an influence of hydrogen bond formation on self-assembly even during fast aggregation. Compared with the randomly scattered, rod-like, self-organized particles of Fp–GABA ester **1** (Figure 4A, with lengths up to 9 μm), the representative SEM images of the solid samples of all the investigated derivatives (Figure 4) revealed very large, hierarchically arranged, supramolecular, round-shaped assemblies of leafy structures, with diameters ranging from 1 to 15 μm in the form of: flowers (Figure 4B, **5,** ≈7 μm), spiral objects (Figure 4C, **8,** ≈15 μm), and artichoke-shaped objects (Figure 4D, **10**, ≈15 μm). The SEM images of all the investigated solid samples are presented in Supporting Information File 1, Figure S2.

The morphological differences of the self-organized fulleropeptide particles and the control compound without the peptide moiety studied here, demonstrate the influence of hydrogen bonds and van der Waals interactions associated with the peptide moiety. This, together with the intermolecular, non-covalent π–π interactions of the fullerene moiety, affects the supramolecular cohesion of fulleropeptide assemblies. The proposed self-assembly path of amphiphilic fulleropeptide esters into spherical particles, their gradual growth to rods or curled leaves, and the final network and artichoke-shaped microstructures are shown in Figure 5.

## Conclusion

The present study could contribute to the further exploration of fulleropeptide esters as self-assembled materials with antioxidant properties. In summary, we have investigated the influence of structurally different subunits on the electrochemical, morphological, and antioxidant properties of previously synthesized fullerene–peptide hybrids. Fulleropeptide esters and non-peptidic fulleropyrrolidine showed mutually, almost identical electrochemical properties with decreased electronegativity in comparison to C_60_. Its hydroperoxide quenching activity for the studied amphiphilic derivatives also remained high. A preliminary in vitro study of the antioxidant efficiency of 12 liposomal fullerene esters, examined by the FOX method, confirmed their much higher antioxidant activity (up to 12-fold) relative to vitamin C. Also, despite the disruption of the π-electron system over a fullerene subunit upon functionalization, similar activity (up to twice better) in relation to C_60_ was reached. Besides improved solubility and high antioxidant capacity, amphiphilic fulleropeptide esters expressed good self-assembly properties, giving ordered structures during both fast aggregation and slow evaporation of the appropriate solvent. In contrast to the rod-like particles of non-peptidic ester **1**, solid samples of fulleropeptides generated different, hierarchical, highly organized assemblies such as flower-like, artichoke-like and spiral objects. During the slow evaporation of the solvent, all compounds, regardless of the number of peptide bonds, spontaneously formed morphologically similar nano- and micro-scale spherical particles, which further assembled into hierarchically more- or less-ordered architectures, resembling circles or networks. Also, the morphology variation ranging from individual spherical to network self-assemblies was achieved by a five-fold concentration increase.

## Experimental

The fullerene–peptide derivatives **2**–**12** were synthesized according to the literature procedure [27]. The solvents (HPLC grade) used for the CV and SEM experiments were stored over 3 Å molecular sieves and degassed under vacuum prior to use. Ferrocene (Fc) and tetrabutylammonium perchlorate (TBAP) were purchased from Sigma-Aldrich and used as received.

### Cyclic voltammetry

The electrochemical behavior of the fullerene esters was investigated using a 1 mM solution in dry DMF, containing 0.1 M TBAP as the supporting electrolyte, in a similar manner to that previously described in [33]. In order to remove oxygen from the electrolyte, the system was bubbled with argon prior to each experiment and the gas remained above the liquid surface during the scans. The electrochemical measurements were carried out on a CHI760b Electrochemical Workstation potentiostat (CH Instruments, Austin, TX) by using a conventional three-electrode cell (5 cm^3^) equipped with a glassy carbon electrode, a silver wire (Ag/Ag^+^) (in contact with 0.01 M AgNO_3_ and 0.10 M TBAP in acetonitrile) and a platinum wire as the working, reference and auxiliary electrodes, respectively, calibrated with a ferrocene/ferrocenyl couple (Fc/Fc^+^) as an internal standard. All experiments were performed at room temperature in the potential range of −3.0 to 0.6 V vs saturated calomel electrode (SCE), with sweep rates between 0.01 and 1 Vs^−1^.

### Scanning electron microscopy

Investigations of sample morphology were carried out with SEM, using a JEOL JSM-840A instrument, at an acceleration voltage of 30 kV. The solid samples were prepared by precipitation from a CHCl_3_/CS_2_ solution with solvents of different polarity (i.e., MeOH, Et_2_O or hexane), and subsequent drying under vacuum. A small amount of each compound was dispersed on a brass substrate. The dried samples obtained from dilute solutions were prepared as follows: 10 μL of 1 mM solution in the PhMe/MeOH (5:1, v/v) mixture of fullerene derivatives was deposited on the surface of a Si substrate (10 × 10 mm) and left overnight to slowly evaporate in a glass petri dish (diameter 10 cm) under PhMe atmosphere at room temperature. Additionally, compounds **9**, **11**, and **12** were prepared in the same solvent system at higher concentration (50 µL of 1 mM solution). The investigated samples were gold sputtered in a JFC 1100 ion sputter device and then subjected to SEM observations.

### In vitro antioxidant activity

The antioxidant activity was determined according to the FOX method [30]. The working FOX reagent was prepared as previously described [25]. The reagent was used within 24 h. The absorbance of the Fe^3+^–XO complex was measured at 560 nm by UV–vis spectrophotometry (GBC-Cintra 40) with 90% MeOH as a zero probe. The detailed procedure of the fullerosome preparation of the tested compound and soybean lecithin in a 1:4 ratio is described in our previous work [25]. The final concentration of the pure 0.002 mg/mL compound was obtained prior to use. The same volume of 200 μM TBHP (obtained by diluting 0.050 mL of 2 mM TBHP with 0.450 mL of H_2_O) was added to the sample and vortexed for 1 min. After 10 min of incubation at room temperature, 0.950 mL of FOX reagent was added to an aliquot of 0.050 mL of the sample. The absorbance at 560 nm was determined for each sample after 80 min of incubation at room temperature. The starting probe of TBHP was prepared in the same manner, replacing the sample by the same volume of H_2_O. The absorbance of the starting probe refers to the starting (maximum) concentration of the peroxide in sample solution, prior to incubation. The difference in the absorbance of the starting probe (*A*_s_) and the sample (*A*) is proportional to the quantity of the peroxide consumed by the sample compound. The blank probe contained 0.950 mL of FOX reagent and 0.050 mL of H_2_O. The absorbance of the blank probe measured at 560 nm (*A*_0_) refers to the colour of the reagent itself in the absence of the peroxide, and all sample and standard absorbance results are normalized by the value of *A*_0_ in the peroxide concentration calculations.

The applicability of the method for use in the range of peroxide concentrations given was confirmed by preparing a standard calibration curve using increasing concentrations of TBHP (0–200 μM) incubated with FOX reagent at room temperature for 30 min. There is a linear relationship between the 560 nm absorbance measurements and the different concentrations of TBHP. All experiments were performed in triplicate, and the average values were taken. The calculation of the direct antioxidant capacity, given as the percentage of consumed TBHP (Δ%), was performed using the following equation:

[1]
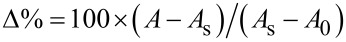


where *A*, *A*_s_ and *A*_0_ represent the absorbance of the tested compound, starting probe (TBHP+FOX) and blank (FOX) probe, respectively. In addition, the relative molar antioxidant capacities (*A*_ox_-rel) of the tested compounds relative to vitamin C are summarized in Table 2.

**Table 2:** The direct antioxidant capacity (Δ%) and the relative antioxidant capacity (*A*_ox_-rel) of the tested compounds (0.002 mg/mL) relative to vitamin C, recalculated to the molar ratio.

Compound	Δ%	*A*_ox_-rel Δ/Δ_vitC_ × *M*/*M*_vitC_

**1**	−23.2	10.2
**2**	−18.6	8.9
**3**	−13.8	7.2
**4**	−17.1	8.0
**5**	−18.0	8.9
**6**	−10.5	5.5
**7**	−18.5	9.4
**8**	−18.5	9.9
**9**	−21.2	11.9
**10**	−19.9	10.9
**11**	−18.1	10.5
**12**	−14.6	8.9
C_60_	−16.7	5.8
Vitamin C	−11.7	1

## Supporting Information

File 1 Additional SEM images of self-organized structures of fullerene esters **1–12** (Figures S1 and S2).
