# Supplementary material for: Fulleropeptide esters as potential self-assembled antioxidants
Source: Beilstein J Nanotechnol. 2015 Apr 27;6:1065–71. doi: 10.3762/bjnano.6.107 (PMC4464194; doi:10.3762/bjnano.6.107)
Supplement: File 1 — Additional SEM images of self-organized structures of fullerene esters 1–12 (Figures S1 and S2). [file Beilstein_J_Nanotechnol-06-1065-s001.pdf]

Supporting Information

for

Fulleropeptide esters as potential self-assembled antioxidants

Mira S. Bjelaković*[1], Tatjana J. Kop[1], Jelena Đorđević[2] and Dragana R. Milić*[2]

Address: [1]Institute of Chemistry, Technology and Metallurgy, Center for Chemistry, University of Belgrade, Njegoševa 12, P.O. Box 473, 11001 Belgrade, Serbia and [2]Faculty of Chemistry, University of Belgrade, Studentski trg 12–16, P.O. Box 51, 11158 Belgrade, Serbia

Email: Mira Bjelaković* - mbjelak@chem.bg.ac.rs, Dragana Milić* - dmilic@chem.bg.ac.rs

* Corresponding author

SEM images of fullerene derivatives 1–12 (Figures S1 and S2)

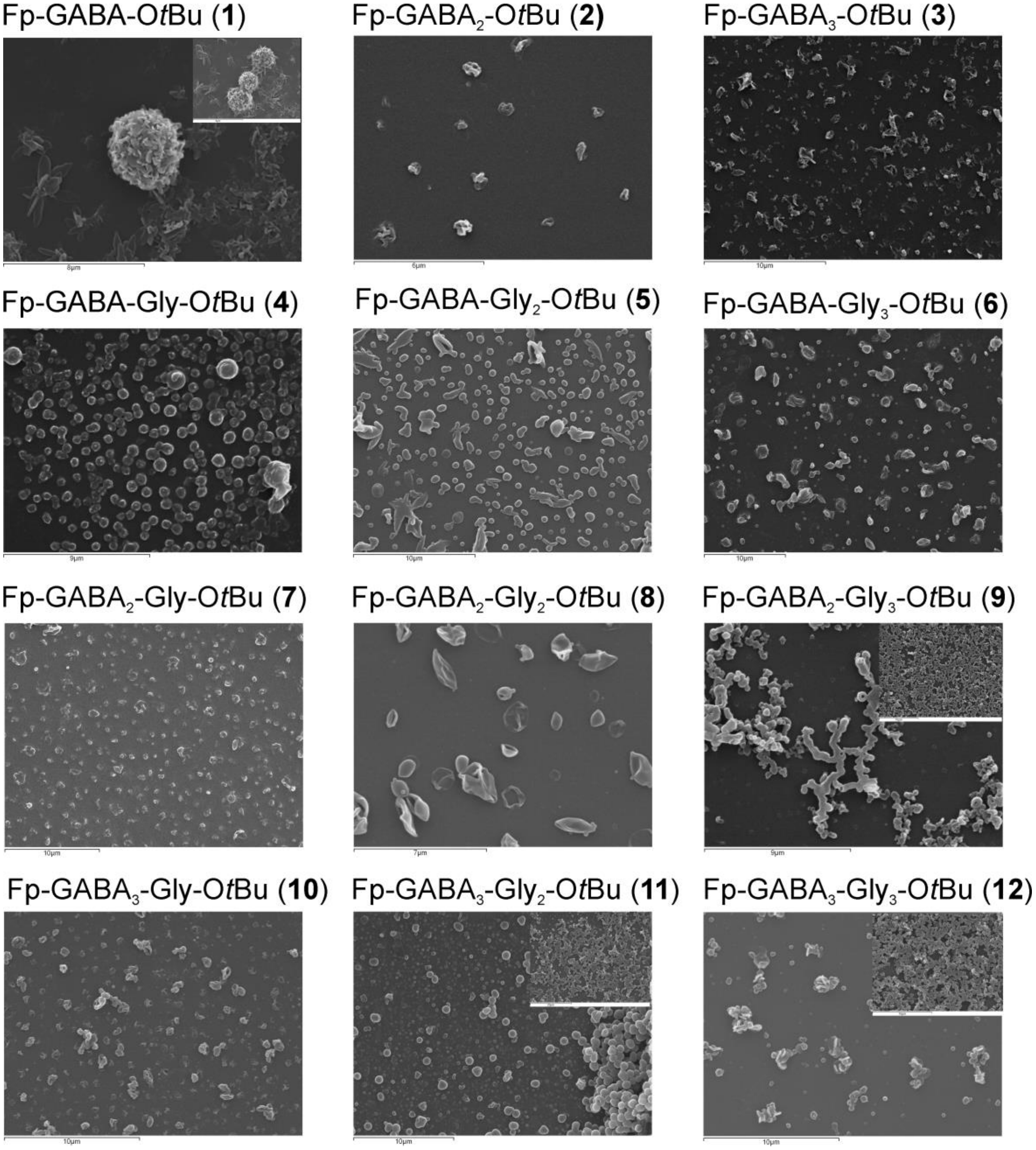

Figure S1: Representative SEM images of the self-organized particles of the parent ester **1** and fulleropeptide esters **2–12** prepared from PhMe/MeOH (5/1, v/v) on Si substrate upon evaporation of 10 μL of 1 mM solution at room temperature; insets on **9**, **11** and **12**: 50 μL of 1 mM solution.

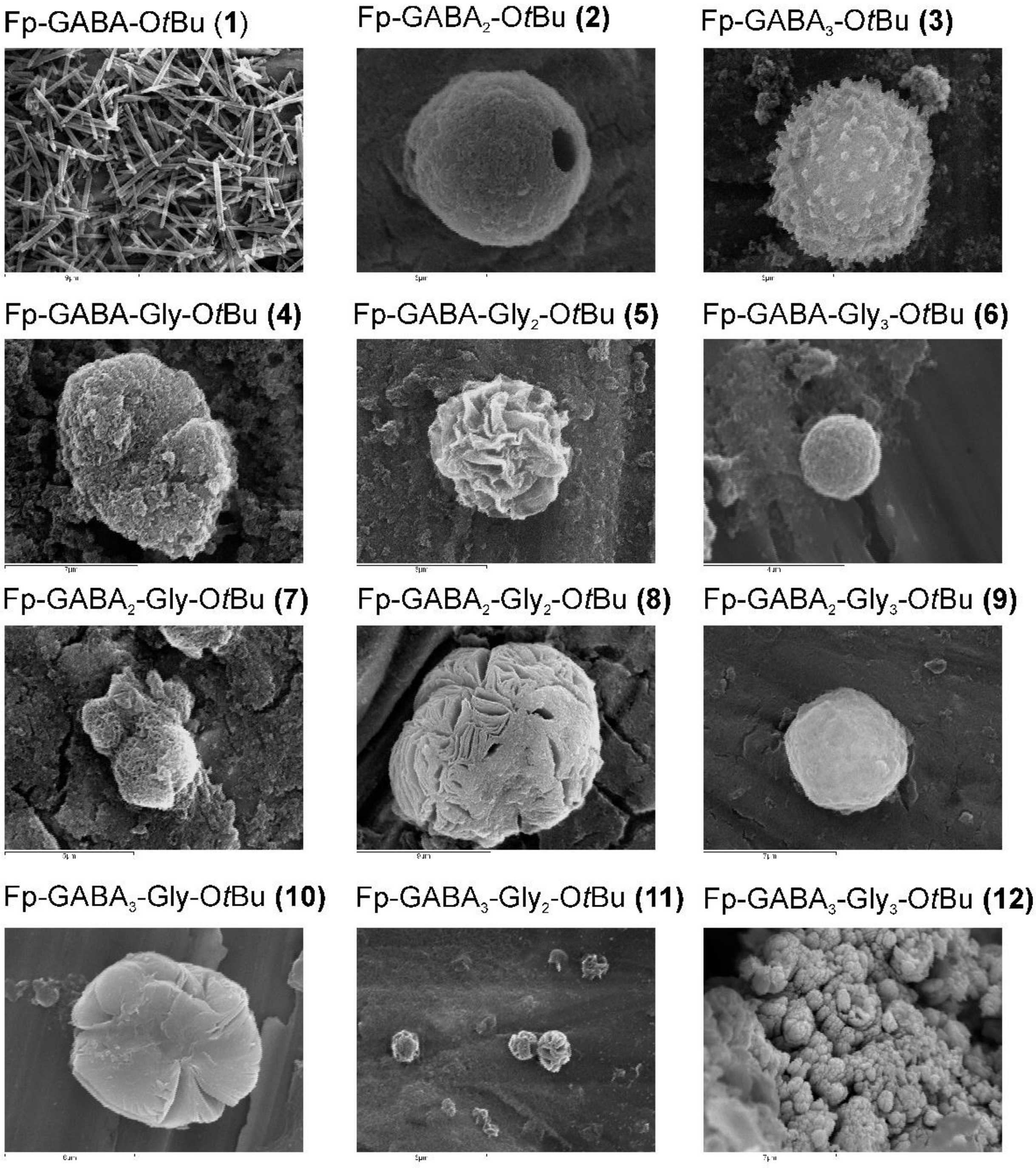

Figure S2: Representative SEM images of the self-organized particles of fulleropyrrolidinic GABA ester **1** and fulleropeptide esters **2–12** prepared from the solids obtained by precipitation with MeOH and deposited on brass substrate.
